# ResearchGate

**DOI:** 10.5195/jmla.2019.643

**Published:** 2019-04-01

**Authors:** Kevin O’Brien

**Affiliations:** Head Librarian, Access to Resources Department, Library of Health Sciences, University of Illinois at Chicago, Chicago, IL, kevinm@uci.edu

Contemporary scholarly scientific research and publishing are characterized by a large number of journals, the fast tempo of publication, and the competitiveness of the funding process. These factors, in conjunction with the pervasive adoption of communication via social media platforms in academia, have given rise to a demand for new venues for scholars and scientists to collaborate on, publicize, share, and quantify the impact of their published works. Because medical librarians are an integral part of the research and scholarly communication process, the popularity of these new platforms calls for a basic familiarity with their features that an informed library professional can provide.

One example of a platform that has emerged in recent years in response to this demand is ResearchGate, a for-profit, social media–like scientific networking and collaboration website. The umbrella term “scholarly collaboration network” has been used to describe platforms like ResearchGate and its competitors.

ResearchGate was founded in Berlin in 2008 by two physicians and a computer scientist. Since its debut, the site has successfully attracted both large numbers of users as well as substantial private investment [1]. ResearchGate claims to have reached the 15 million member mark in 2017 [2].

ResearchGate’s primary feature is the individual researcher profile, which is used to promote scholarly production. The site creates profiles with information harvested from literature databases and other sources, while permitting researchers to create profiles by registering on the site. Standard elements of a profile include a dashboard-like overview, citations to published work, contact and career information, research interests, links to citations of potential interest, and selected impact metrics. Profiles can be augmented by including contact information, a photograph, citations to work that has not been discovered by ResearchGate, and full-text article content for sharing with other members. Site members can follow other researchers and their work, identify colleagues and coworkers such as lab personnel, and share details of current projects.

One distinctive feature is a question submission-and-response knowledgebase, allowing members to pose, respond to, and track questions regarding research and other topics of interest. There is also a proprietary quantitative altmetric called RG score. This score is based on work appearing in the researcher profile and other ResearchGate members’ interactions with it. The RG score has attracted criticism aimed at a lack of transparency in how it is calculated and at vulnerabilities leading to the potential of intentional inflation by those seeking to abuse it [3].

Revenue streams for the website include advertising that appears on its question-and-answer database page, job recruitment listings, and conference announcements. These displays are customized for individual users.

One characteristic ResearchGate shares with social media platforms is vigorous user engagement activity. The site frequently generates emails encouraging members to log in to monitor how many new views their profiles have garnered, how many members are following their research, and other metrics of engagement. While such notification messages can be managed in member account settings, these persistent enticements to spend time on the site mimic aspects of social media and are drawing increasing amounts of criticism. As with other social media platforms, the potential for misuse and malicious exposure of the accumulated user data are also concerns.

The high visibility that ResearchGate has achieved has not come without controversy. During the platform’s rise to prominence, one factor in its popularity was the large volume of full-text portable document format (PDF) articles present in many researcher profiles. These full-text PDFs were easily discoverable in web searches, making ResearchGate a popular source for article sharing.

Relevant to note is that a sizeable percentage of the articles that were available on ResearchGate were versions of PDFs that were protected by copyright law and not permitted to be shared. This fact came to the attention of a number of publishers and resulted in a coordinated effort on their part to address this issue.

In 2017, a group of publishers, including such large firms as ACS Publications and Elsevier, formed an organization called the Coalition for Responsible Sharing to pressure ResearchGate to take measures against distributing copyright-protected material on its platform. The coalition advocated for adherence to the International Association of Scientific, Technical and Medical (STM) Publishers’ “Voluntary Principles for Article Sharing on Scholarly Collaborations Networks,” a document outlining parameters for approved sharing among researchers [4].

ResearchGate responded to this pressure by removing some copyright-protected content, but at the time of this writing, the issue had not been completely resolved. Several large publishers, including SpringerNature, have recently announced an agreement to explore ways to allow their content to be shared legally on ResearchGate [5]. ACS and Elsevier are pursuing the matter in a US federal court [6].

ResearchGate’s success in building a large user base gives it the potential to survive the substantial legal challenges it faces. While the platform’s scale and attractive user interface may appeal to many researchers, issues such as a lack of transparency in the composition of the RG score, concerns regarding use of member data, and an attitude of ambivalence toward the complicated topic of article sharing contribute to a strong case that ResearchGate is not the optimal solution to the pressing need for a space for scholars and scientists to freely collaborate and communicate regarding their work.
